# *NOTCH3* variants are more common than expected in the general population and associated with stroke and vascular dementia: an analysis of 200 000 participants

**DOI:** 10.1136/jnnp-2020-325838

**Published:** 2021-03-12

**Authors:** Bernard P H Cho, Stefania Nannoni, Eric L Harshfield, Daniel Tozer, Stefan Gräf, Steven Bell, Hugh S Markus

**Affiliations:** 1 Department of Clinical Neurosciences, University of Cambridge, Cambridge, Cambridgeshire, UK; 2 Department of Medicine, University of Cambridge, Cambridge, Cambridgeshire, UK

## Abstract

**Background:**

Cysteine-altering *NOTCH3* variants identical to those causing the rare monogenic form of stroke, CADASIL (cerebral autosomal dominant arteriopathy with subcortical infarcts and leukoencephalopathy), have been reported more common than expected in the general population, but their clinical significance and contribution to stroke and dementia risk in the community remain unclear.

**Methods:**

Cysteine-altering *NOTCH3* variants were identified in UK Biobank whole-exome sequencing data (N=200 632). Frequency of stroke, vascular dementia and other clinical features of CADASIL, and MRI white matter hyperintensity volume were compared between variant carriers and non-carriers. MRIs from those with variants were visually rated, each matched with three controls.

**Results:**

Of 200 632 participants with exome sequencing data available, 443 (~1 in 450) carried 67 different cysteine-altering *NOTCH3* variants. After adjustment for various covariates, *NOTCH3* variant carriers had increased risk of stroke (OR: 2.33, p=0.0004) and vascular dementia (OR: 5.00, p=0.007), and increased white matter hyperintensity volume (standardised difference: 0.52, p<0.001) and white matter ultrastructural damage on diffusion MRI (standardised difference: 0.72, p<0.001). On visual analysis of MRIs from 47 carriers and 148 matched controls, variants were associated with presence of lacunes (OR: 5.97, p<0.001) and cerebral microbleeds (OR: 4.38, p<0.001). White matter hyperintensity prevalence was most increased in the anterior temporal lobes (OR: 7.65, p<0.001) and external capsule (OR: 13.32, p<0.001).

**Conclusions:**

Cysteine-changing *NOTCH3* variants are more common in the general population than expected from CADASIL prevalence and are risk factors for apparently ‘sporadic’ stroke and vascular dementia. They are associated with MRI changes of small vessel disease, in a distribution similar to that seen in CADASIL.

## Introduction

Cerebral small vessel disease (SVD) is a major cause of stroke and dementia.^1 2^ Most cases are sporadic, although polygenic risk factors appear important.^3^ SVD is the most common type of ischaemic stroke caused by single-gene disorders; the most common is cerebral autosomal dominant arteriopathy with subcortical infarcts and leukoencephalopathy (CADASIL), which results in early-onset lacunar stroke and dementia, and is caused by distinctive *NOTCH3* variants that lead to an extra unpaired cysteine residue in one of 34 epidermal growth factor-like repeat (EGFR) domains.^4^

Although CADASIL is considered rare, with a reported disease prevalence of 4 per 100 000,^5 6^ a much higher frequency of typical cysteine-altering *NOTCH3* variants was reported as 1 in 400 individuals.^7 8^ These could contribute to the risk of apparently sporadic lacunar stroke, but the clinical significance of these variants remains uncertain. Studies have mostly been conducted on anonymised genome-sequencing databases,^7^ and it is unclear whether such apparently ‘asymptomatic’ variants have clinical implications.^9^ It has been demonstrated that such variants are more likely to be in EGFR domains 7–34, while variants within clinical CADASIL cases are more likely, although not exclusively, in domains 1–6.^10^

To determine whether these variants increase disease, analysis of sequencing databases in which clinical data are also available is required. We used whole-exome sequence data from 200 632 participants in UK Biobank to determine the frequency of *NOTCH3* variants, and whether they were associated with stroke and dementia. Moreover, in the subset of individuals in whom MRI scanning was performed, we correlated presence of *NOTCH3* variants with MRI markers of SVD including white matter hyperintensities (WMHs) and lacunar infarcts and determined whether variant carriers had a similar spatial distribution of lesions to that seen in typical CADASIL cases. This study extends previous studies, particularly the Geisinger DiscovEHR cohort,^8^ with a larger sample size providing greater power to examine associations with the risk of both stroke and dementia.

## Methods

### Study population

UK Biobank is a prospective study of over 500 000 healthy volunteers aged 40–69 years recruited across the UK between 2006 and 2010.^11^ About 9 million individuals were invited to join, of whom 5.5% participated in the baseline assessment.^12^ Phenotypic data were collected through questionnaires and physical examinations. A subset of 100 000 individuals are also participating in the MRI Study, and approximately 40 000 brain MRIs were available at the time of this analysis. These participants were selected on the basis of travelling distance from the imaging centre and not clinical information. All MRIs were performed on one of the two identical Skyra 3.0T scanners (Siemens Medical Solutions, Germany). Identical acquisition parameters and quality control was used for all scans.^13^ In October 2020, whole-exome sequences of 200 632 UK Biobank participants were released and all were included here. This analysis was performed under UK Biobank application number 36509.

### Ascertainment of *NOTCH3* pathogenic variants

The variants located in the *NOTCH3* gene (chromosome 19:15,159,038-15,200,995, reference genome assembly GRCh38) were extracted from the exome data in PLINK format. The extracted variants were annotated using Ensembl Variant Effect Predictor.^14^ Missense variants that lead to the gain or loss of a cysteine residue in one of the 34 EGFR domains of the NOTCH3 protein (amino acid position 40-1373, Uniprot accession number: Q9UM47) were identified.

### Phenotypic data fields

History of vascular risk factors, measured blood pressure, blood pressure medication and parental history of stroke were recorded. History of diseases, including stroke, vascular dementia, epilepsy, depression, migraine and myocardial infarction, were determined from self-report, hospital and death records (code list in online supplemental table 1). The Framingham cardiovascular risk score was calculated.^15^ Results of cognitive tests,^16^ including pairs matching, reaction time, prospective memory, fluid intelligence, numeric memory, trail-making test part B and the symbol digit substitution test, were extracted and converted into z-scores. The average of all z-scores was used as an estimate of global cognitive function.^17^ The following blood tests were extracted (cholesterol, low-density lipoprotein, high-density lipoprotein (HDL), apolipoprotein A, apolipoprotein B, lipoprotein A (Lp(a)), triglycerides, glycated haemoglobin, glucose, C reactive protein (CRP), creatinine, cystatin C and urea).

10.1136/jnnp-2020-325838.supp1 Supplementary data

### Brain imaging analysis

In the 19 686 participants with MRI available, measures were compared between variant subjects and controls. UK Biobank neuroimaging working group-derived MRI measures for brain volume and WMH volume were used, generated by an image-processing pipeline developed and run on behalf of UK Biobank.^13^ Brain volume was estimated by SIENAX,^18^ and normalised for head size. WMHs were quantified on fluid attenuated inversion recovery (FLAIR) images through the brain intensity abnormality classification algorithm.^19^ Furthermore, the peak width skeletonised mean diffusivity (PSMD)^20^ was derived in-house from diffusion tensor imaging (DTI) data and is a marker of diffuse white matter damage.^19^

Additionally, visual review of MRI scans (T1-weighted, T2 FLAIR and susceptibility-weighted imaging (SWI)) from those cases with *NOTCH3* variants and MRI available (N=47) was performed by a neurologist (SN). Each variant case was matched with three controls for age, sex, ethnicity and family history of stroke. Scans were analysed blinded to subject identity. Lacunar infarcts, cerebral microbleeds, and the spatial distribution and extent of WMH were quantified. Lacunes were counted on FLAIR images, defined as a round or ovoid subcortical fluid-filled cavity of 3–15 mm in diameter in the territory of a single perforating arteriole.^21^ Cerebral microbleeds were counted using the brain observer microbleed scale,^22^ defined as round well-defined hypointense foci on SWI with a diameter of 2–10 mm. The spatial distribution and extent of WMH in different brain regions was quantified on T2 FLAIR images using the modified Schelten’s scale.^23^

### Calculation of polygenic risk score

To compare the risk conferred by *NOTCH3* variants with that by common stroke variants, we calculated an ischaemic stroke polygenic risk score (PRS) using more than 3 million common genetic variants.^24^ We performed linear regression of the PRS adjusting for the first 10 ancestry principal components in UK Biobank to account for known differences in PRS performance across ethnic groups.^25^ Standardised residuals from this regression model were used for analyses. We calculated the OR for ischaemic stroke in *NOTCH3* carriers compared with non-carriers and the OR for a 1 SD increase in the ischaemic stroke PRS, dividing the former by the latter (on the log-scale, assuming a linear association) to estimate the increment of the PRS in SD that was predicted to be equivalent to the risk conferred by *NOTCH3* variants.^26^ To examine whether the ischaemic stroke PRS affected *NOTCH3* penetrance, we performed statistical tests for interaction between the PRS and *NOTCH3* status; we divided our sample cohort into three groups based on their PRS: low (bottom 20% of PRS), intermediate and high (top 20%) risk.^27^

### Statistical analysis

The effect of *NOTCH3* variants on phenotype was assessed by linear regression for continuous outcomes and logistic regression for binary outcomes. For the former, effect sizes were standardised to enable a more informative comparison. Firth’s correction was applied to all logistic regression models to account for rare event bias.^28^ All regression models were adjusted for age, sex, exome sequencing batch and first 10 principal components of ancestry. Any data fields that did not follow a normal distribution were transformed through: natural log (HDL, triglycerides, CRP and WMH), inverse normalisation (Lp(a), Framingham cardiovascular risk score and the regional WMH scores) or square root (cerebral microbleed and lacune counts). When comparing demographic and risk factor profiles between variant and non-variant groups, Pearson’s Χ^2^ test was used for categorical variables and the two-sample t-test was used for continuous variables. All statistical analyses were performed using R V.3.6.2 and Stata V.15.1 with 2-sided p values and p<0.05 for statistical significance.

## Results

### The prevalence of *NOTCH3* pathogenic variants

Of the 200 632 participants with exome sequencing data, 443 (2.2 per 1000) carried *NOTCH3* variants leading to an unpaired cysteine residue in one of the 34 EGFR domains of the NOTCH3 protein. All carriers were heterozygotes, giving a population frequency of 1 in 452, about 100-fold higher than expected based on CADASIL prevalence estimations of 2–5 in 100 000.^7^

### Distribution of *NOTCH3* pathogenic variants

**Figure 1:**
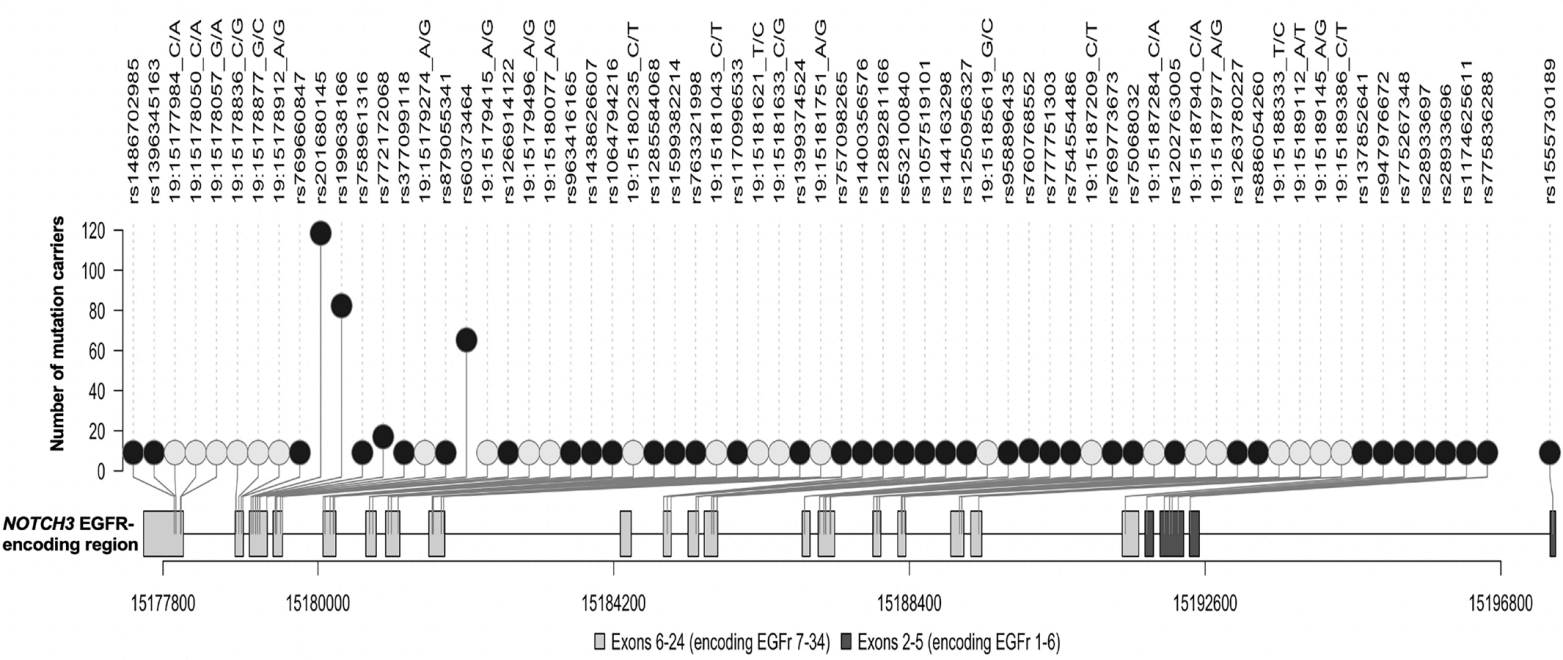
Lolliplot showing the distribution of distinct pathogenic variants in UK Biobank across the reverse strand of the *NOTCH3* gene. Lighter circles represent variants without rsID; darker circles represent variants with rsID; lighter rectangular boxes represent exons that encode EGFRs 7–34; darker rectangular boxes represent exons that encode EGFRs 1–6. EGFR, epidermal growth factor-like repeat.

The distribution of variants is shown in figure 1, online supplemental table 2 and online supplemental figure 1. In total, 67 unique *NOTCH3* pathogenic variants were identified, of which 36 were observed in only one individual. The identified variants were spread across 22 different exons between exons 2 and 24 of the *NOTCH3* gene (except exon 16; figure 1). They affected 31 EGFR domains, involving all EGFR domains except 7, 21 and 34. Variants were predominantly located towards the distal end of NOTCH3 protein; only 11 participants had a *NOTCH3* variant located in EGFR domains 1–6 (figure 1 and online supplemental figure 1). More than half of the variants (39 of 67, 58.2%) involved the swapping of cysteine and arginine residues.

Sixty-five different variants were found in 378 white participants, 3 in 41 individuals of Asian or Asian British ancestry, 3 in 7 Black or Black British participants, 1 variant in a Chinese participant, 1 in 4 mixed ethnicity individuals and 4 in other ethnic groups. Ten of the unique variants occurred in non-white subjects, and three were not observed in white individuals (p.Cys579Tyr, p.Gly861Cys and p.Cys1250Arg).

### *NOTCH3* variants were not associated with vascular risk factors, blood biochemistry or cognition

The demographic and risk factor profile of cases with and without variants are shown in table 1. There were no significant differences between groups, except that the variant group had more men and less white subjects. *NOTCH3* variants were not associated with vascular risk factors, blood biochemistry markers, the Framingham cardiovascular risk score or cognitive function (online supplemental figure 2).

**Table 1:** Comparison of the demographic and risk factor profile for cases with and without *NOTCH3* variants

	*NOTCH3* variant carriers	Non-*NOTCH3* variant carriers	P value
Number of cases	443	200 189	
Mean (SD) age at recruitment (years)	55.7 (8.2), range 40–70	56.5 (8.2) range 38–72	0.06
Male sex (%)	223 (50)	89 872 (45)	0.02
White ethnic background (%)	378 (85)	187 912 (94)	1.53×10^–15^
Mean (SD) BMI (kg/m^2^)	27.8 (5.0)	27.4 (4.8)	0.12
Non-smoker (%)	399 (90.0)	180 955 (90.4)	0.82
Blood pressure medication (%)	37 (8.4)	20 171 (10.1)	0.23
Hypertension (%)	142 (32.1)	65 361 (32.6)	0.79
Hypercholesterolaemia (%)	49 (11.1)	19 085 (9.5)	0.27
Diabetes mellitus (%)	41 (9.3)	14 257 (7.1)	0.08

The p values for categorical and continuous variables were calculated by the Pearson’s Χ^2^ test and two-sample t-test, respectively.

BMI, body mass index.

### *NOTCH3* variant carriers were at an increased risk of stroke and vascular dementia

**Figure 2:**
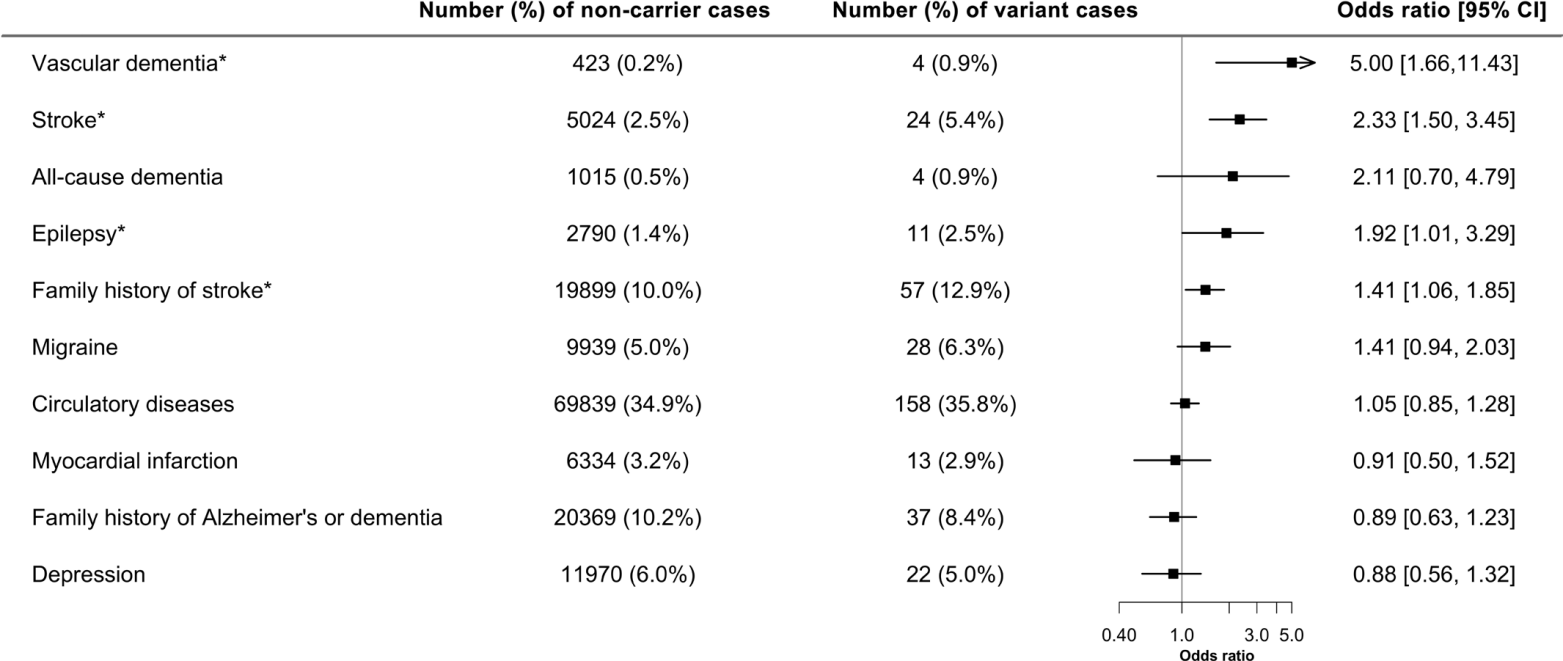
Forest plot showing the association of *NOTCH3* variant with different diagnoses. Family history and prevalence of disease cases were analysed with the presence of *NOTCH3* variant through logistic regression. Firth’s correction was applied to all the regression models. N=200 632. *Significance at p<0.05.

Presence of *NOTCH3* variants was associated with a twofold increase in the odds of stroke (OR: 2.33, 95% CI: 1.50 to 3.45, p=0.0004). Vascular dementia (OR: 5.00, 95% CI: 1.66 to 11.43, p=0.007) was also more frequent in variant carriers. All dementia cases in *NOTCH3* carriers were of vascular origin (and occurred after enrolment into UK Biobank). All-cause dementia was not significantly associated with the presence of *NOTCH3* variants (OR: 2.11, 95% CI: 0.70 to 4.79, p=0.16). Population attributable fractions showed that 0.26% of stroke and 0.72% vascular dementia in UK Biobank were attributed to the *NOTCH3* variants. There was a borderline significant increase in epilepsy (OR:1.92, 95% CI: 1.01 to 3.29, p=0.048). Presence of a variant was associated with an increased family history of stroke (OR:1.41, 95% CI: 1.06 to 1.85, p=0.02). No significant associations were found with migraine (OR: 1.41, 95% CI: 0.94 to 2.03, p=0.09) or depression (OR: 0.88, 95% CI: 0.56 to 1.32, p=0.56) (figure 2).

Significant associations were repeated after additional adjustment for diabetes, hypertension and smoking. Results were unaltered for vascular dementia (OR: 5.00, 95% CI: 1.66 to 11.48, p=0.007), stroke (OR: 2.37, 95% CI: 1.52 to 3.53, p=0.0003) and family history of stroke (OR: 1.42, 95% CI: 1.06 to 1.86, p=0.02).

**Figure 3:**
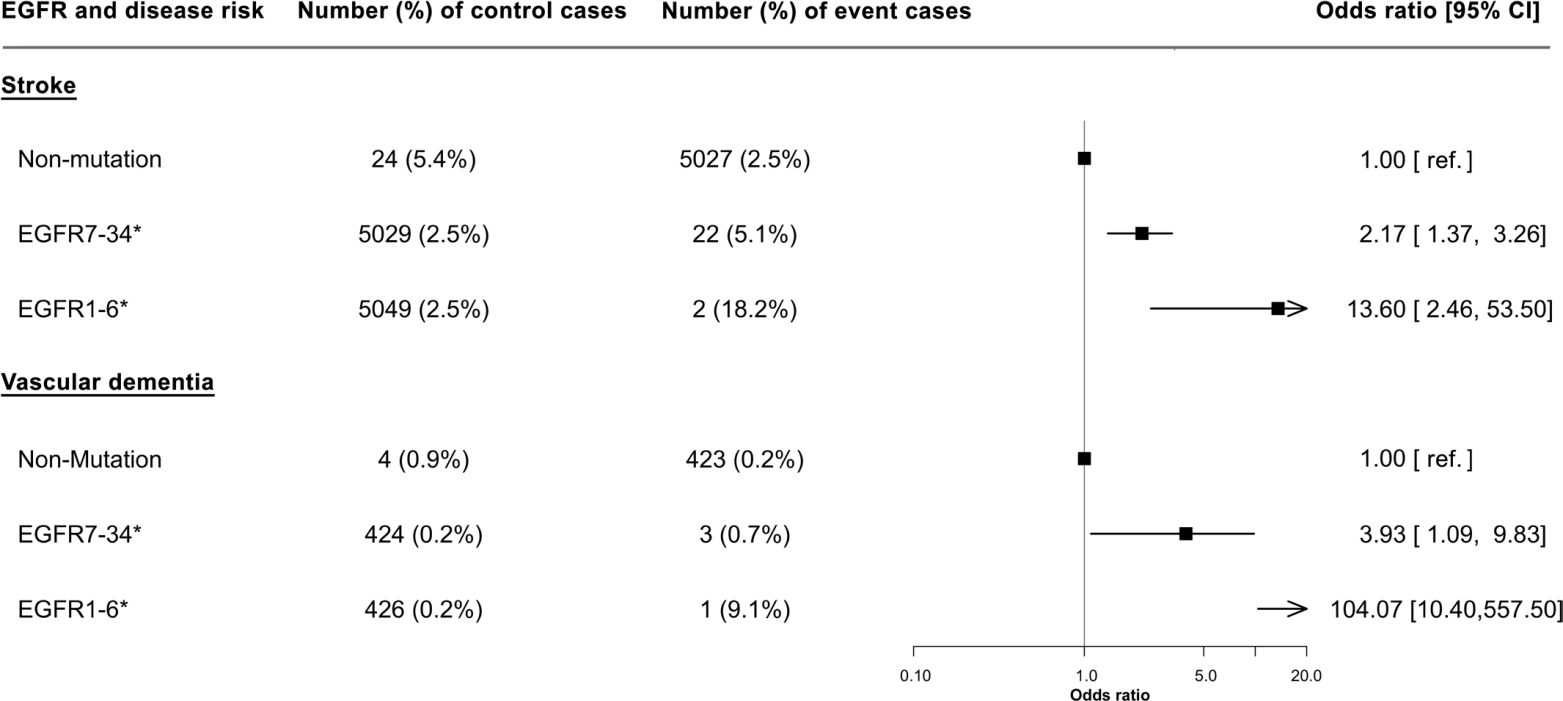
Forest plot showing the effect of *NOTCH3* variant location on the odds of stroke or vascular dementia. The location of variant was stratified by EGFRs 7–34 and EGFRs 1–6; their effect on disease risk was relative to that without the variant. Firth’s correction was applied to all the regression models. N=200 632. *Significance at p<0.05. EGFR, epidermal growth factor-like repeat.

The risk of both stroke and vascular dementia was higher with variants in EGFRs 1–6 compared with EGFRs 7–34 (figure 3). The risk of stroke was 13-fold higher (OR 13.60, 95% CI: 2.46 to 53.50, p=0.006) and overdouble (OR 2.17, 95% CI: 1.37 to 3.26, p=0.001) than that of variant-free individuals, respectively, for variants in EGFRs 1–6 and 7–34, with findings for vascular dementia risk mirroring this.

Similar associations were observed when the same analyses were performed for incident cases of outcomes, that is, those that had occurred from the point of recruitment into UK Biobank to the latest follow-up in March 2019 (online supplemental figure 3, 4).

### *NOTCH3* variant carriers showed signs of brain MRI abnormalities

*NOTCH3* variants were associated with an increase in WMH volume (standardised difference: 0.52, 95% CI: 0.29 to 0.75, p=9.1×10^−6^) and DTI-PSMD (standardised difference: 0.72, 95% CI: 0.39 to 1.03, p=7.0×10^−8^) but not with brain volume.

**Figure 4:**
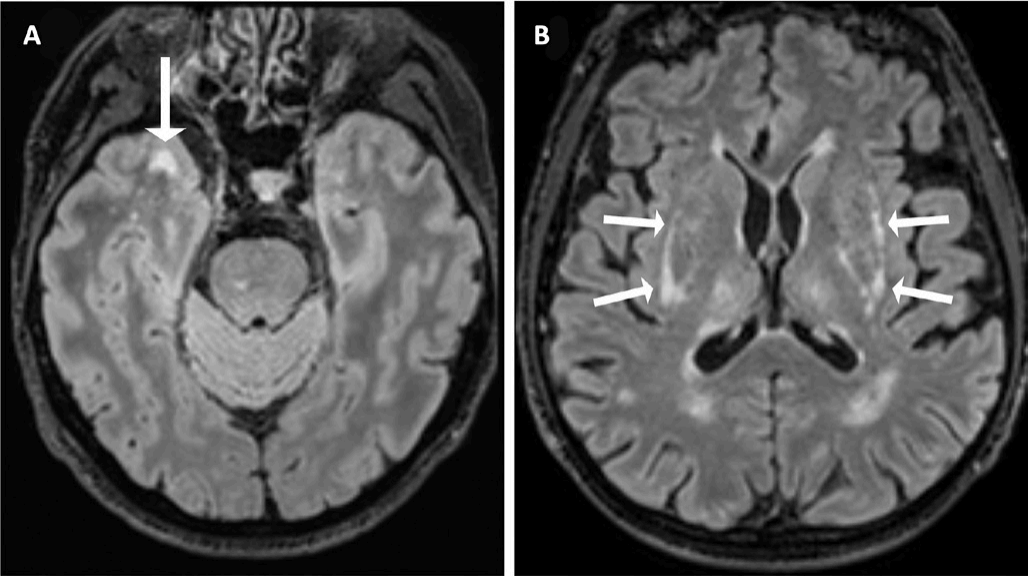
Axial T2 FLAIR brain MRI of two subjects with a pathogenic *NOTCH3* variant in UK Biobank. (A) A woman in her 50s, carrying a p.Arg1231Cys variant that affects EGFR domain 31, with WMH involving the right anterior temporal lobe (arrow). (B) A man in his 60s, carrying a p.Arg578Cys variant that affects EGFR domain 14, with WMH involving the external capsule bilaterally (arrows). EGFR, epidermal growth factor-like repeat; FLAIR, fluid attenuated inversion recovery; WMH, white matter hyperintensity.

MRI scans from 47 *NOTCH3* variant carriers and 148 matched controls were visually rated and compared (table 2, figure 4 and online supplemental figure 5). Image sequences available were T1-weighted (47 cases, 148 controls), T2 FLAIR (45 cases, 148 controls) and SWI (44 cases, 148 controls). *NOTCH3* variants were associated with an increased risk of the presence of lacunes (OR: 5.97, 95% CI: 2.00 to 12.06, p=0.0002) and cerebral microbleeds (OR: 4.38, 95% CI: 1.69 to 7.76, p=0.0003). There were also significant association with the number of lacunes (standardised difference: 0.59, 95% CI: 0.23 to 0.89, p=0.0007) and borderline association with the number of cerebral microbleeds (standardised difference: 0.37, 95% CI: 0.05 to 0.74, p=0.046).

**Table 2:** Comparison of WMH distribution on the modified Schelten’s scale between *NOTCH3* variant carriers and non-carriers

Brain regions	Number (%) of non-carrier cases	Number (%) of mutation cases	OR (95% CI)	P value
Periventricular white matter	108 (73)	36 (80)	1.51 (0.65 to 3.88)	0.34
Frontal white matter	119 (80)	40 (89)	1.40 (0.50 to 4.57)	0.53
Parietal white matter	79 (53)	34 (76)	2.44 (1.11 to 5.68)	0.03
Occipital white matter	45 (30)	20 (44)	1.82 (0.88 to 3.80)	0.11
Anterior temporal white matter	12 (9)	17 (38)	7.65 (3.14 to 19.81)	<0.001
Posterior temporal white matter	45 (30)	14 (31)	0.94 (0.42 to 2.03)	0.87
Corpus callosum	7 (5)	5 (11)	2.34 (0.65 to 7.95)	0.18
Caudate	1 (1)	3 (7)	7.80 (1.30 to 72.60)	0.03
Putamen	16 (11)	9 (20)	1.97 (0.77 to 4.88)	0.15
Globus pallidus	4 (3)	0 (0)	0.19 (0.00 to 2.13)	0.21
Thalamus	4 (3)	6 (13)	3.90 (1.05 to 14.91)	0.04
Internal capsule	3 (2)	2 (4)	2.20 (0.35 to 11.12)	0.37
External capsule	9 (6)	17 (38)	13.32 (4.96 to 39.89)	<0.001
Cerebellum	4 (3)	3 (7)	2.10 (0.43 to 8.91)	0.34
Mesencephalon	3 (2)	2 (4)	3.84 (0.57 to 27.84)	0.16
Pons	25 (17)	11 (24)	1.51 (0.64 to 3.46)	0.34
Medulla	2 (1)	0 (0)	0.80 (0.01 to 8.86)	0.87

Table showing the effect of the presence of *NOTCH3* variants on the odds of developing any WMH in each brain region.

WMH, white matter hyperintensity.

### *NOTCH3* variants were associated with a CADASIL-like distribution of WMH

**Figure 5:**
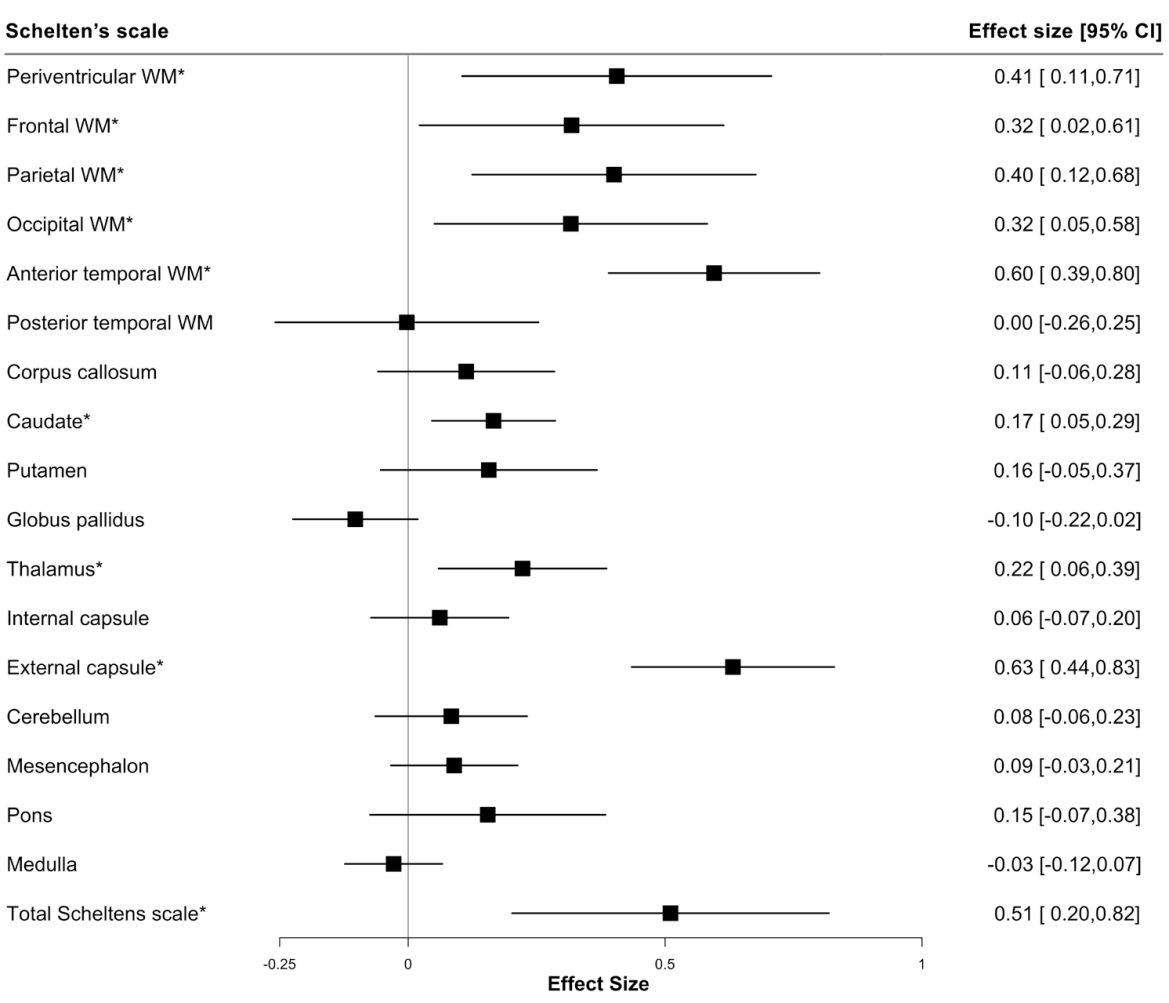
Forest plot showing the severity of WMH in different brain regions. Inversely transformed measures of the Schelten’s scale were analysed with the presence of *NOTCH3* variant through linear regression. A total of 193 participants were included in the regression analyses, of which 45 were variant carriers and 148 were non-carriers. *Significance p<0.05. WM, white matter; WMH, white matter hyperintensity.

The presence of *NOTCH3* variants was correlated with the spatial distribution and extent of WMH using the modified Schelten’s scale. First, we examined the distribution of the presence of any WMH in each brain region (table 2). *NOTCH3* variants were most strongly associated with any WMH in two areas previously reported to be predilection sites for WMH in CADASIL,^25^ the anterior temporal lobe (OR: 7.65, 95% CI: 3.14 to 19.81, p<0.001) and external capsule (OR: 13.32, 95% CI: 4.96 to 39.89, p<0.001). Typical example scans are shown in figure 4. Second, we examined the severity of WMH in each region, as assessed on the Schelten’s scale. *NOTCH3* variants were associated with higher aggregate WMH score (standardised difference: 0.51, 95% CI: 0.24 to 0.84, p=0.001) and higher individual scores for 8 of the 17 brain regions examined, including the periventricular region, frontal lobe, parietal lobe, occipital lobe, anterior temporal lobe, caudate, thalamus and external capsule (figure 5). Concordantly, the differences between variant carriers and non-carriers were most marked for the anterior temporal lobe and the external capsule. All these associations remained significant after accounting for multiple comparisons using a false-discovery rate of 5% with the Benjamini-Hochberg procedure.

### *NOTCH3* associations with ischaemic stroke were independent of polygenic risk

**Figure 6:**
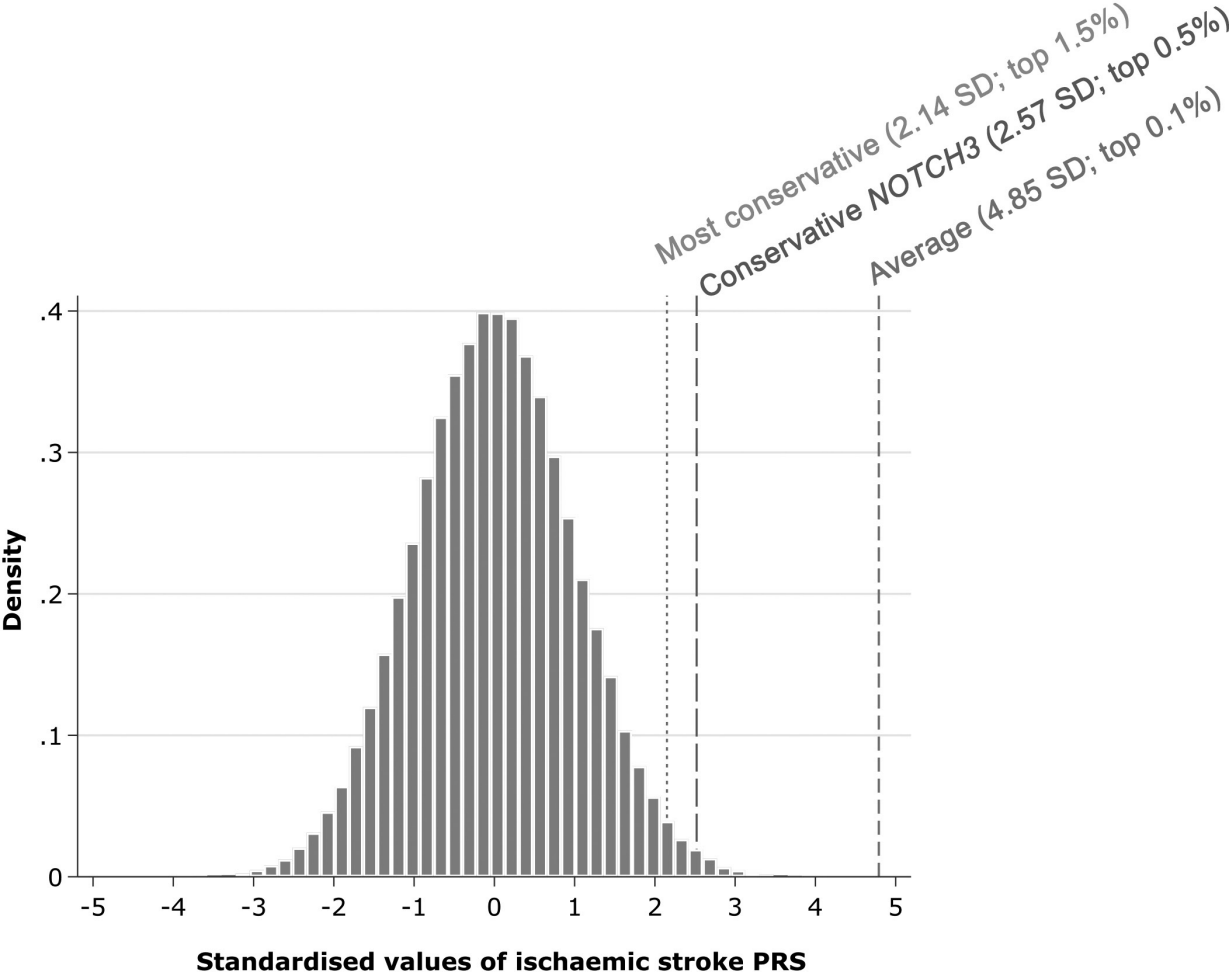
Estimated polygenic risk equivalent for the association of *NOTCH3* status with ischaemic stroke. Average=ratio of the log-OR of models of *NOTCH3* status and a 1 SD increase in polygenic risk score (PRS) for ischaemic stroke; conservative *NOTCH3*=ratio of the lower 95% CI of the log-OR for *NOTCH3* status and the log-OR for a 1 SD increase in PRS for ischaemic stroke; most conservative=ratio of the lower 95% CI of the log-OR for *NOTCH3* status and the upper 95% CI of the log-OR for a 1 SD increase in PRS for ischaemic stroke. Population percentages calculated using a 1-sided z-score.

*NOTCH3* carriers had a threefold increased risk of ischaemic stroke (OR 3.07, 95% CI: 1.81 to 5.23, p<0.001), while a 1 SD increase in ischaemic stroke PRS increased the odds by 26% (OR: 1.26, 95% CI: 1.21 to 1.32, p<0.001). We calculated presence of a *NOTCH3* variant therefore conferred the same risk as a 4.85 SD increase in PRS (figure 6). Conscious of substantial uncertainty in our estimated association for *NOTCH3* status and ischaemic stroke, we calculated more conservative estimates using the lower bound of 95% CI for *NOTCH3* with the OR (conservative *NOTCH3*) as well as upper bound of the 95% CI for the association with the PRS (most conservative). These placed carriers of *NOTCH3* variants as having a polygenic risk equivalent to the upper 1.5% of the population. We observed no interaction between *NOTCH3* carrier status and PRS (online supplemental figure 6).

## Discussion

In this study of 200 632 individuals, we show that *NOTCH3* variants are much more common than expected in the general population, being present in 1 in 450 individuals. We demonstrated that these ‘asymptomatic’ variants are associated with an increased risk of both stroke and vascular dementia, and with MRI features of SVD, including lacunar infarcts, WMH, diffuse white matter damage on DTI and cerebral microbleeds. Taken together, these results suggest that typical CADASIL variants are common in the general population, and account for a significant burden of apparently ‘sporadic’ stroke and dementia.

CADASIL is reported to be rare, with an estimated disease prevalence of 4 per 100 000 individuals in UK populations.^5 6^ In contrast, recent studies have demonstrated an increased frequency of cysteine-changing *NOTCH3* variants in population databases,^7 9^ but these have been anonymised databases making it impossible to determine whether these variants associate with clinical disease. A recent report from the US Geisinger database suggested *NOTCH3* variants are associated with an increased risk of stroke and MRI features of SVD, although no association was found with dementia.^8^ Our findings are broadly confirmatory of those in the Geisinger cohort although we reported a slightly higher prevalence of *NOTCH3* variants (0.22% vs 0.14%) and our larger sample size also allowed an association with vascular dementia to be detected.

Our findings provide robust evidence from a large population-based study of over 200 000 individuals that common *NOTCH3* variants are associated with symptomatic cerebrovascular disease in the general population. We demonstrated that they conferred a risk equivalent to that in the top 1.5% of individuals as determined by a PRS, with the risks from common polygenic variant and *NOTCH3* variants being independent. Of note, the contribution of variants causing familial stroke to the risk of common stroke is being increasingly recognised, not only in the context of CADASIL, but for example in moyamoya disease.^29^

In addition to an association with stroke, we demonstrate a strong association with vascular dementia. All cases of dementia occurring in patients with *NOTCH3* variants were of a vascular origin; no association was found with all-cause dementia, reflecting the fact that many cases of dementia in older individuals are due to non-vascular pathologies. We also found trends towards other clinical features of CADASIL being more common with these variants with a marginally significant association with epilepsy, and a non-significant trend towards increased risk of migraine.

In addition to looking at overall brain MRI measures, we analysed the distribution of white matter lesions in individuals with *NOTCH3* variants, compared with matched controls. The greatest increase in WMH was in two areas known to be particularly affected in CADASIL, namely the anterior temporal lobes and external capsule.^30^ This suggests that the *NOTCH3* variants are not merely associated with stroke, but also with a specific CADASIL-like phenotype in the general population. Although the phenotype of the variant carriers is much milder in the UK Biobank cohort, the distribution of white matter changes is similar to that seen in patients with CADASIL.

It remains uncertain why some patients with *NOTCH3* variants develop the CADASIL phenotype, while others appear asymptomatic. One emerging factor is variant position. Within CADASIL cohorts, variants in EGFRs 1–6 have been associated with earlier stroke onset than variants in EGFRs 7–34.^10^ In previous population studies, variants in EGFRs 7–34 were much more common,^10^ and we confirmed this in UK Biobank. Furthermore, we found that although variants in EGFRs 1–6 were associated with higher risk of stroke and vascular dementia in the general population, more distal variants in EGFRs 7–34 were still associated with risk of both conditions. CADASIL variants alter the extracellular domain of *NOTCH3* (Notch3^ECD^) resulting in its multimerisation and aberrant accumulation in granular osmiophilic material.^31^ The mutant Notch3^ECD^ facilitates interactions with components of the cerebrovascular extracellular matrix, including the metalloproteinase inhibitor TIMP3 and vitronectin, and promotes their accumulation and sequestration in Notch3^ECD^-containing deposits. How this process is affected by *NOTCH3* pathogenic variant position is unclear; it could result from differences in the expression, processing, interaction and aggregation properties of mutant *NOTCH3*. Nonetheless, variant site fails to account for much of the phenotypic heterogeneity. Vascular risk factors, particularly smoking and hypertension, have been shown to be associated with an earlier onset of stroke within CADASIL families but only account for a small amount of variability.^32 33^ Family studies have suggested that as much of 60% of the heritability of WMH lesion volume is accounted for by yet undetermined modifier genetic factors outside the *NOTCH3* gene, but the nature of these remains undetermined.^34^

Our study has a number of strengths. First, it included data from over 200 000 individuals with exome sequencing. Second, we correlated the genotyping with extensive clinical information, including prospectively collected information obtained from linked electronic health records. This allowed us to identify associations not only with disease prevalence but also incident disease occurring during prospective follow-up. This revealed similar associations with stroke and vascular dementia to that observed in the prevalence data. Third, all scans had been performed on one of the two identical MR scanners using identical sequences improving consistency and quality.

Our study also has limitations. Diseases like dementia can sometimes be misclassified or under-represented by the ICD codes in health records. Our analysis was in predominantly white populations. CADASIL has been shown to be present in all ethnic groups in which it is being looked for, and we have no reason to suspect the associations reported are different in other ethnic groups. However, frequency of *NOTCH3* variants may differ between ethnic groups, and initial data suggest that it is higher in Far Eastern populations including Taiwan.^35^ Pertinently, a recent report suggested typical CADASIL variants may predispose to multifactorial late-onset stroke in Taiwan.^36^

In conclusion, our study shows that typical cysteine-changing *NOTCH3* variants are common in the general population and these variants are associated with increased risk of both stroke and vascular dementia, and with MRI markers of SVD including WMH and lacunar infarcts. This demonstrates that genetic variation in the *NOTCH3* gene accounts for a much greater proportion of stroke in the general population than previously thought. Although the magnitude of risk for a *NOTCH3* variant on stroke and vascular dementia partly depends on variant site, modifiers outside the *NOTCH3* gene appear to be important. Further research is required to identify these. Finally, our study emphasises that the boundaries between monogenic and polygenic stroke are not as distinct as previously believed.

## Data Availability

Data are available in a public, open access repository. Data from UK Biobank (https://www.ukbiobank.ac.uk/) are available to bona fide researchers on application. This study was performed under UK Biobank application number 36509.
