# Supplementary material for: NOTCH3 variants are more common than expected in the general population and associated with stroke and vascular dementia: an analysis of 200 000 participants
Source: J Neurol Neurosurg Psychiatry. 2021 Mar 12;92(7):694–701. doi: 10.1136/jnnp-2020-325838 (PMC8223663; doi:10.1136/jnnp-2020-325838)
Supplement: Supplementary data [file jnnp-2020-325838supp001.pdf]

SUPPLEMENTAL MATERIAL
(2 tables and 6 figures)

Supplementary table 1. The code list used for the retrieval of disease records.

Biobank Code Text	Code type	Code	Date of last linkage
Stroke	UK Biobank algorithmically defined stroke	42007	March 2019
	UK Biobank self-report	Field 20002 Codes 1081,1086,1491,1583	May 2020
	ICD9 for hospital and death record retrieval	430, 431, 434, 436	May 2020
	ICD10 for hospital and death record retrieval	I60, I61, I63, I64	May 2020
Family history of stroke	Illness of father	Field 20107 Code 2	May 2020
	Illness of mother	Field 20110 Code 2	May 2020
All-cause dementia	UK Biobank algorithmically defined dementia	42019	March 2019
Vascular dementia	UK Biobank algorithmically defined vascular dementia	42023	March 2019
	ICD9 for hospital and death record retrieval	290.2,2903,2904,2912,2941,3310,3311,3312, 3315	May 2020
	ICD10 for hospital and death record retrieval	F00, F01, F02, F03, G30, A81.0, F05.1,F10.6,G31.0,G31.1,G31.8,I67.3	May 2020
Family history of Alzhiemer's or dementia	Illness of father	Field 20107 Code 10	May 2020
	Illness of mother	Field 20110 Code 10	May 2020
Migraine	UK Biobank algorithmically defined migraine	131053	March 2019
	UK Biobank self-report	Field 20002 Code 1265	May 2020
	ICD9 for hospital record retrieval	346	May 2020
	ICD10 for hospital record retrieval	G43	May 2020
Epilepsy	UK Biobank algorithmically defined epilepsy	131049	March 2019
	UK Biobank self-report	Field 20002 Code 1264	May 2020
	ICD9 for hospital and death record retrieval	345	May 2020
	ICD10 for hospital and death record retrieval	G40	May 2020
Depression	Touchscreen Q&A	Fields 20124 and 20125	May 2020
	ICD9 for hospital record retrieval	311	May 2020
	ICD10 for hospital record retrieval	F32,F33	May 2020
Myocardial infarction	UK Biobank algorithmically defined myocardial infarction	42001	March 2019
	UK Biobank self-report	Field 20002 Code 1075	May 2020
	ICD9 for hospital and death record retrieval	410, 411, 412, 436	May 2020
	ICD10 for hospital and death record retrieval	I21, I22, I23, I24.1,I25.2	May 2020
Circulatory diseases	ICD9 for hospital record retrieval	39, 40, 41, 42, 43,44, 45	May 2020
	ICD10 for hospital record retrieval	I0, I1, I2, I3, I4, I5, I6, I7, I8, I9	May 2020

Cho BPH, *et al. J Neurol Neurosurg Psychiatry* 2021; 92:694–701. doi: 10.1136/jnnp-2020-325838

Supplementary table 2. Sixty-seven distinct cysteine altering *NOTCH3* variants identified in UK Biobank. All the variants lead to an uneven number of cysteine residues in the EGFR domains of the NOTCH3 protein.

Protein change	Genomic change	Codon change	Exon	EGFR domain	Frequency in UK Biobank	Ethnicities of variant carriers in UK Biobank
p.Arg54Cys	g.15197537G>A	c.160C>T	2	1	1	White (1)
p.Arg110Cys	g.15192389G>A	c.328C>T	3	2	1	White (1)
p.Arg141Cys	g.15192218G>A	c.421C>T	4	3	3	White (3)
p.Arg169Cys	g.15192134G>A	c.505C>T	4	4	1	White (1)
p.Arg182Cys	g.15192095G>A	c.544C>T	4	4	3	White (3)
p.Arg207Cys	g.15192020G>A	c.619C>T	4	5	1	White (1)
p.Tyr258Cys	g.15191774T>C	c.773A>G	5	6	1	Others (1)
p.Arg332Cys	g.15191466G>A	c.994C>T	6	8	1	White (1)
p.Cys360Tyr	g.15189386C>T	c.1079G>A	7	9	1	White (1)
p.Cys408Arg	g.15189145A>G	c.1222T>C	8	10	1	White (1)
p.Cys419Ser	g.15189112A>T	c.1255T>A	8	10	1	White (1)
p.Tyr465Cys	g.15188333T>C	c.1394A>G	9	11	1	White (1)
p.Ser476Cys	g.15188301T>A	c.1426A>T	9	12	2	White (2)
p.Gly481Cys	g.15188286C>A	c.1441G>T	9	12	1	White (1)
p.Cys504Arg	g.15187977A>G	c.1510T>C	10	12	1	White (1)
p.Cys516Phe	g.15187940C>A	c.1547G>T	10	13	6	White (6)
p.Arg532Cys	g.15187893G>A	c.1594C>T	10	13	2	White (2)
p.Cys554Phe	g.15187284C>A	c.1661G>T	11	14	1	White (1)
p.Arg558Cys	g.15187273G>A	c.1672C>T	11	14	1	White (1)
p.Arg578Cys	g.15187213G>A	c.1732C>T	11	14	7	White (6), Asian or Asian British (1)
p.Cys579Tyr	g.15187209C>T	c.1736G>A	11	14	1	Black or Black British (1)
p.Arg587Cys	g.15187186G>A	c.1759C>T	11	15	4	White (3), Black or Black British (1)
p.Arg607Cys	g.15187126G>A	c.1819C>T	11	15	4	White (4)
p.Arg640Cys	g.15186911G>A	c.1918C>T	12	16	13	White (8), Asian or Asian British (1), Chinese (1), Others (3)
p.Cys654Tyr	g.15185670C>T	c.1961G>A	13	16	1	White (1)
p.Ser671Cys	g.15185619G>C	c.2012C>G	13	17	2	White (2)
p.Arg680Cys	g.15185593G>A	c.2038C>T	13	17	1	White (1)
p.Arg717Cys	g.15185404G>A	c.2149C>T	14	18	11	White (11)
p.Arg728Cys	g.15185371G>A	c.2182C>T	14	18	5	White (5)
p.Arg767Cys	g.15185017G>A	c.2299C>T	15	19	5	White (5)
p.Arg785Cys	g.15184963G>A	c.2353C>T	15	20	7	White (7)

p.Trp802Cys	g.15184910C>A	c.2406G>T	15	20	3	White (3)
p.Gly861Cys	g.15181787C>A	c.2581G>T	17	22	1	Black or Black British (1)
p.Cys873Arg	g.15181751A>G	c.2617T>C	17	22	1	White (1)
p.Cys910Tyr	g.15181639C>T	c.2729G>A	17	23	2	White (2)
p.Cys912Ser	g.15181634A>T	c.2734T>A	17	23	2	White (2)
p.Tyr916Cys	g.15181621T>C	c.2747A>G	17	23	3	White (3)
p.Cys939Ser	g.15181140A>T	c.2815T>A	18	24	2	White (1)
p.Cys971Tyr	g.15181043C>T	c.2912G>A	18	25	1	White (1)
p.Cys986Arg	g.15180999A>G	c.2956T>C	18	25	1	White (1)
p.Cys1015Arg	g.15180780A>G	c.3043T>C	19	26	1	White (1)
p.Arg1031Cys	g.15180732G>A	c.3091C>T	19	26	3	White (3)
p.Cys1055Tyr	g.15180235C>T	c.3164G>A	20	27	1	White (1)
p.Cys1061Tyr	g.15180217C>T	c.3182G>A	20	27	1	White (1)
p.Arg1076Cys	g.15180173G>A	c.3226C>T	20	27	1	White (1)
p.Arg1100Cys	g.15180101G>A	c.3298C>T	20	28	1	White (1)
p.Cys1108Arg	g.15180077A>G	c.3322T>C	20	28	1	White (1)
p.Cys1110Arg	g.15179496A>G	c.3328T>C	21	28	2	White (2)
p.Cys1119Tyr	g.15179468C>T	c.3356G>A	21	28	2	White (2)
p.Cys1137Arg	g.15179415A>G	c.3409T>C	21	29	1	White (1)
p.Arg1143Cys	g.15179397G>A	c.3427C>T	21	29	68	White (66), Black or Black British (2)
p.Tyr1144Cys	g.15179153T>C	c.3431A>G	21	29	2	White (2)
p.Cys1157Arg	g.15179274A>G	c.3469T>C	22	29	1	White (1)
p.Arg1190Cys	g.15179175G>A	c.3568C>T	22	30	9	White (9)
p.Arg1201Cys	g.15179142G>A	c.3601C>T	22	30	20	White (19), Black or Black British (1)
p.Arg1210Cys	g.15179115G>A	c.3628C>T	22	31	2	White (1), Others (1)
p.Cys1222Gly	g.15179079A>C	c.3664T>G	22	31	85	White (85)
p.Arg1231Cys	g.15179052G>A	c.3691C>T	22	31	121	White (72), Mixed (4), Asian or Asian British (38), Black or Black British (1), Others (6)
p.Arg1242Cys	g.15178936G>A	c.3724C>T	23	31	5	White (5)
p.Cys1250Arg	g.15178912A>G	c.3748T>C	23	32	1	Asian or Asian British (1)
p.Cys1261Trp	g.15178877G>C	c.3783C>G	23	32	1	White (1)
p.Cys1275Ser	g.15178837A>T	c.3823T>A	23	32	1	White (1)
p.Arg1291Cys	g.15178057G>A	c.3871C>T	24	33	1	White (1)
p.Cys1293Phe	g.15178050C>A	c.3878G>T	24	33	1	White (1)
p.Cys1315Trp	g.15177983G>C	c.3945C>G	24	33	1	White (2)
p.Cys1315Phe	g.15177984C>A	c.3944G>T	24	33	2	White (1)
p.Cys1324Ser	g.15177958A>T	c.3970T>A	24	33	1	White (1)

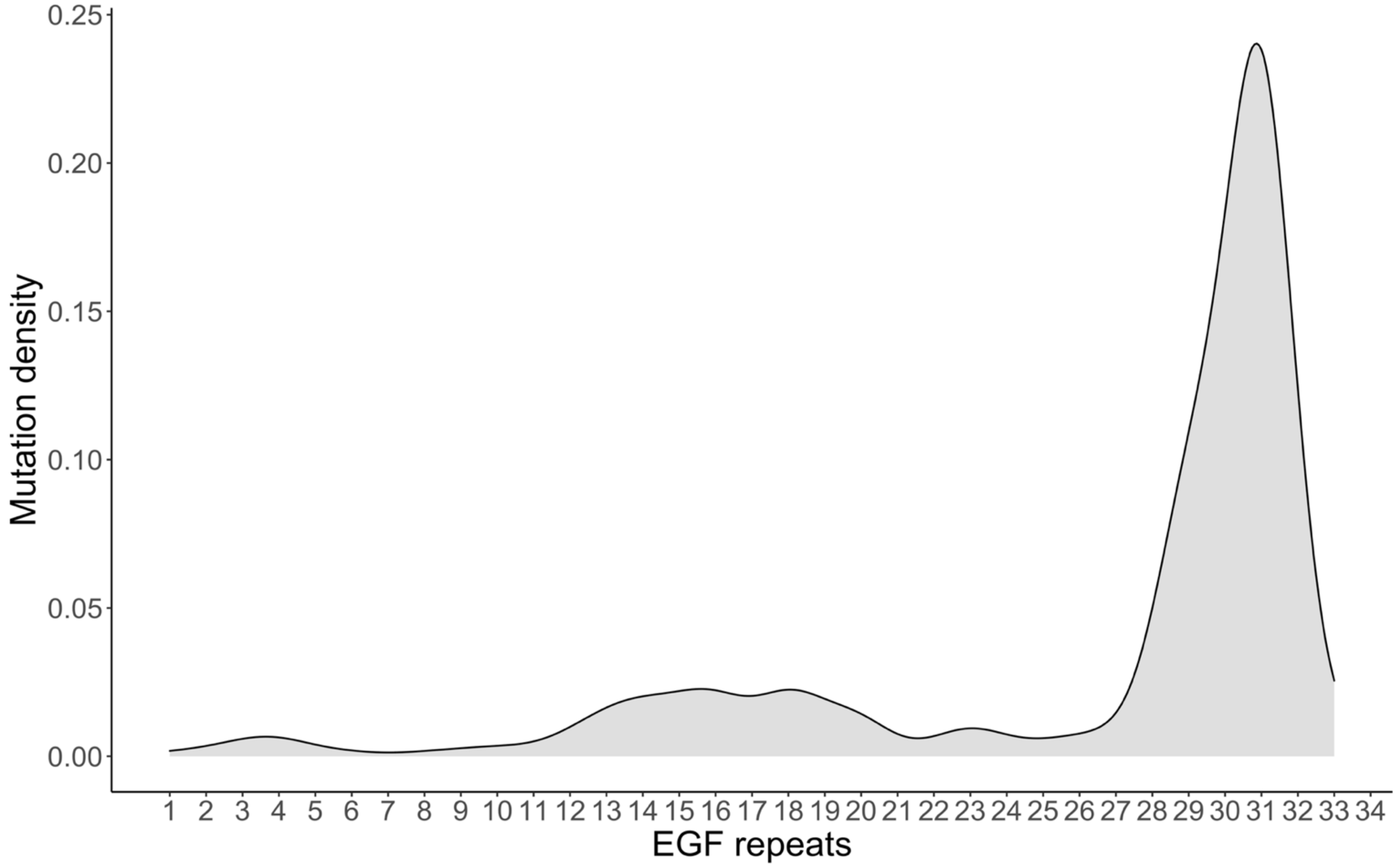

Supplementary figure 1. Density plot showing the distribution of variants across the 34 EGFR domains of *NOTCH3*

Cho BPH, *et al. J Neurol Neurosurg Psychiatry* 2021; 92:694–701. doi: 10.1136/jnnp-2020-325838

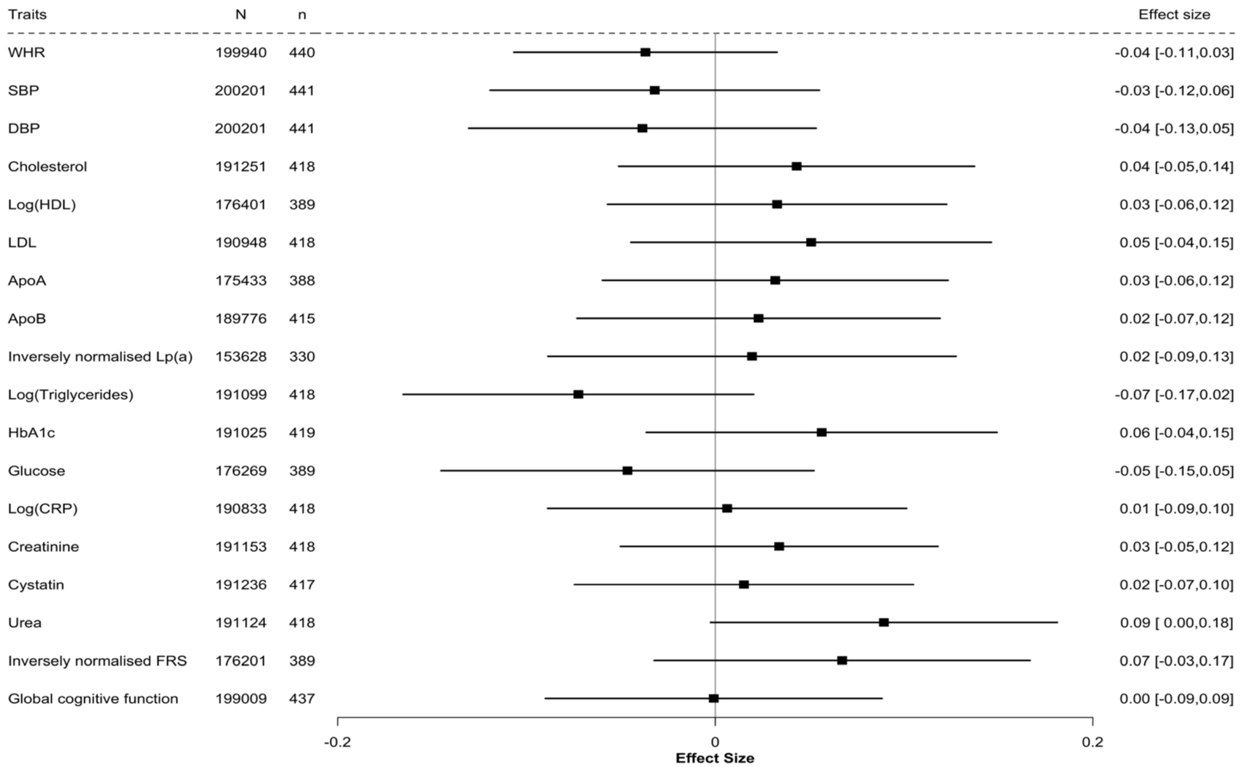

Supplementary figure 2. Forest plot showing the standardised effect of *NOTCH3* variant on different clinical traits. Measurements of blood pressure, blood biochemistry, Framingham risk score (FRS) and cognitive function were analysed with the presence of *NOTCH3* variant through linear regression. N, total number of participants included in regression analysis; n, number of variant carriers included in the analysis; CI, confidence interval; bp, blood pressure; HDL, high-density lipoprotein; LDL, low-density lipoprotein; ApoA, apolipoprotein a; ApoB, apolipoprotein b; Lpa, lipoprotein a; HbA1c, glycated haemoglobin.

Disease incidence	Number (%) of non-carrier cases	Number (%) of variant cases	Odds ratio [95% CI]
Vascular dementia*	189 (0.09%)	4 (0.9%)	11.08 [3.64,25.64]
Epilepsy*	693 (0.3%)	6 (1.4%)	4.41 [1.81, 8.83]
Stroke*	2001 (1.0%)	12 (2.7%)	2.97 [1.60, 5.03]
All-cause dementia	925 (0.5%)	4 (0.9%)	2.32 [0.77, 5.27]

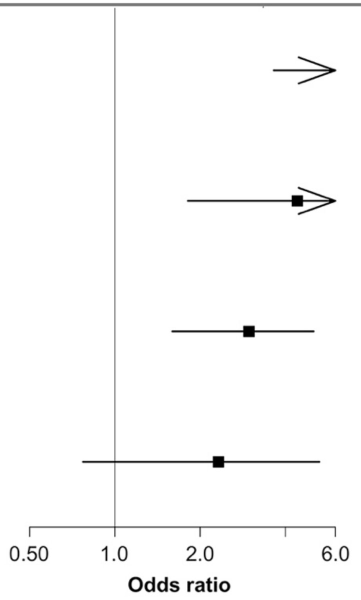

Supplementary figure 3. Forest plot showing the effect of *NOTCH3* variants on the odds of incident cases of stroke, all-cause dementia, vascular dementia or epilepsy. Firth's correction was applied to all the logistic regression models. Total number of participants included in the regression analyses (N) = 200,632. CI, confidence interval; *, significance at $p < 0.05$.

EGFR and disease incidence	Number (%) of control cases	Number (%) of event cases	Odds ratio [95% CI]
**Stroke**			
Non-mutation	12 (2.8%)	2002 (1.0%)	1.00 [ref.]
EGFR7-34*	2002 (1.0%)	12 (2.8%)	3.04 [1.63, 5.14]
EGFR1-6	2014 (1.0%)	0 (0.0%)	5.25 [0.04, 43.92]
**Vascular dementia**			
Non-Mutation	4 (0.9%)	189 (0.09%)	1.00 [ref.]
EGFR7-34*	190 (0.09%)	3 (0.7%)	8.69 [2.40, 21.98]
EGFR1-6*	192 (0.1%)	1 (9.1%)	252.89 [25.17,1374.12]

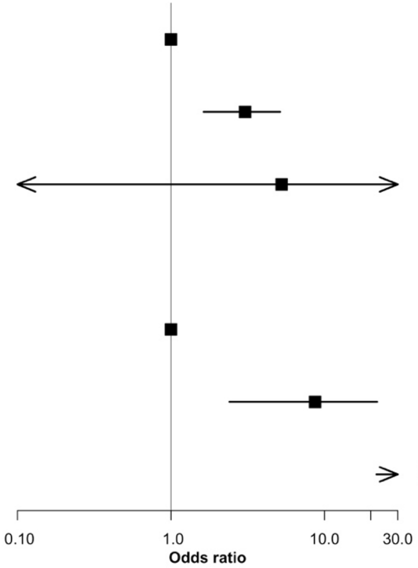

Supplementary figure 4. Forest plot showing the effect of *NOTCH3* variant location on the odds of stroke or vascular dementia cases from the point of recruitment to last follow-up. The location of variant was stratified by EGFR 7-34 and EGFR 1-6; their effect on disease risk was relative to that without the variant. Firth's correction was applied to all the regression models. Total number of participants included in the regression analyses (N) = 200,632. CI, confidence interval; *, significance at $p < 0.05$.

Cho BPH, *et al. J Neurol Neurosurg Psychiatry* 2021; 92:694–701. doi: 10.1136/jnnp-2020-325838

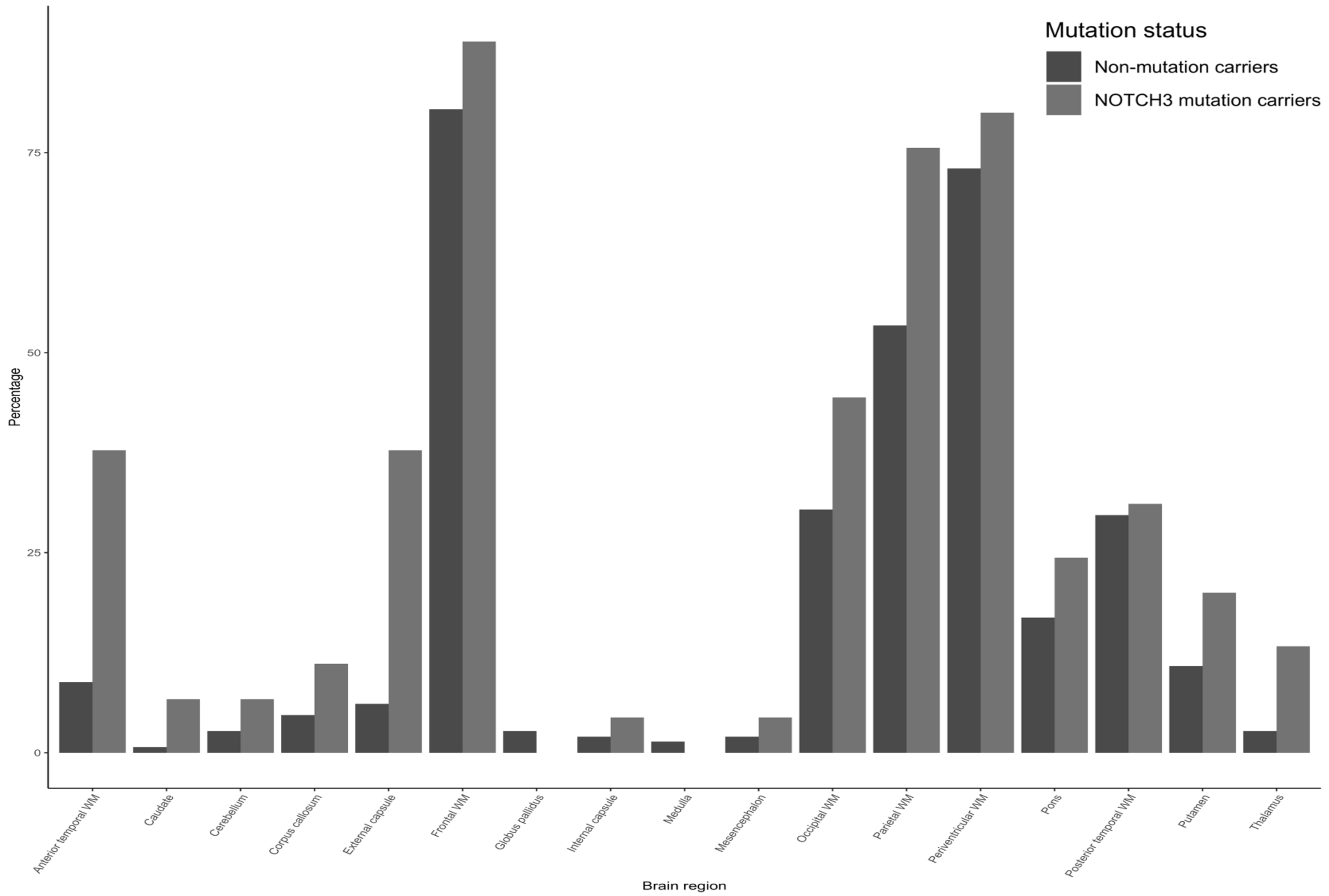

Supplementary figure 5. Bar chart showing the proportion of participants who had any WMH present in the each of the brain regions.

Cho BPH, *et al. J Neurol Neurosurg Psychiatry* 2021; 92:694–701. doi: 10.1136/jnnp-2020-325838

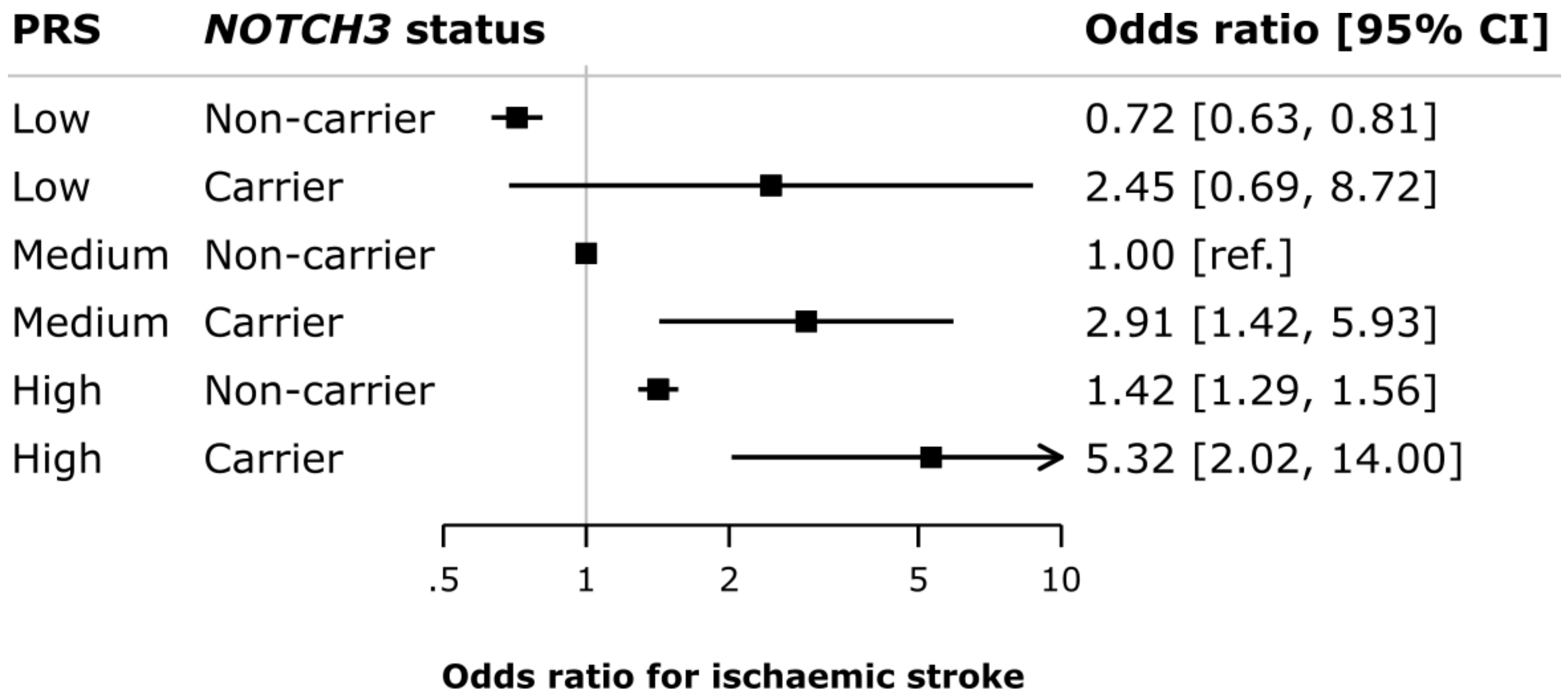

Supplementary Figure 6. Forest plot displaying the odds ratios and associated 95% confidence intervals for ischaemic stroke stratified by polygenic risk score and *NOTCH3* status.

P-value for a multiplicative continuous interaction with standardised score = 0.86; p-value for a multiplicative interaction with the categorical low, intermediate and high PRS groupings = 0.95; p-value for the additive interaction between continuous PRS assessed using the relative excess risk due to interaction (RERI) = 0.48.

Cho BPH, *et al. J Neurol Neurosurg Psychiatry* 2021; 92:694–701. doi: 10.1136/jnnp-2020-325838
